# Colony-stimulating factor 1 receptor blockade prevents fractionated whole-brain irradiation-induced memory deficits

**DOI:** 10.1186/s12974-016-0671-y

**Published:** 2016-08-30

**Authors:** Xi Feng, Timothy D. Jopson, Maria Serena Paladini, Sharon Liu, Brian L. West, Nalin Gupta, Susanna Rosi

**Affiliations:** 1 Brain and Spinal Injury Center, University of California, 1001 Potrero Ave, Bldg. 1, Room 101, San Francisco, CA 94110 USA; 2 Department of Physical Therapy and Rehabilitation Science, University of California, San Francisco, CA USA; 3 Department of Neurological Surgery, University of California, San Francisco, CA USA; 4 Plexxikon Inc, Berkeley, CA USA; 5 Department of Pediatrics, University of California, San Francisco, CA USA

**Keywords:** CSF-1R, Whole-brain irradiation, Cognition

## Abstract

**Background:**

Primary central nervous system (CNS) neoplasms and brain metastases are routinely treated with whole-brain radiation. Long-term survival occurs in many patients, but their quality of life is severely affected by the development of cognitive deficits, and there is no treatment to prevent these adverse effects. Neuroinflammation, associated with activation of brain-resident microglia and infiltrating monocytes, plays a pivotal role in loss of neurological function and has been shown to be associated with acute and long-term effects of brain irradiation. Colony-stimulating factor 1 receptor (CSF-1R) signaling is essential for the survival and differentiation of microglia and monocytes. Here, we tested the effects of CSF-1R blockade by PLX5622 on cognitive function in mice treated with three fractions of 3.3 Gy whole-brain irradiation.

**Methods:**

Young adult C57BL/6J mice were given three fractions of 3.3 Gy whole-brain irradiation while they were on diet supplemented with PLX5622, and the effects on periphery monocyte accumulation, microglia numbers, and neuronal functions were assessed.

**Results:**

The mice developed hippocampal-dependent cognitive deficits at 1 and 3 months after they received fractionated whole-brain irradiation. The impaired cognitive function correlated with increased number of periphery monocyte accumulation in the CNS and decreased dendritic spine density in hippocampal granule neurons. PLX5622 treatment caused temporary reduction of microglia numbers, inhibited monocyte accumulation in the brain, and prevented radiation-induced cognitive deficits.

**Conclusions:**

Blockade of CSF-1R by PLX5622 prevents fractionated whole-brain irradiation-induced memory deficits. Therapeutic targeting of CSF-1R may provide a new avenue for protection from radiation-induced memory deficits.

**Electronic supplementary material:**

The online version of this article (doi:10.1186/s12974-016-0671-y) contains supplementary material, which is available to authorized users.

## Background

Whole-brain irradiation (WBI) is commonly used for the treatment of primary brain tumors and brain metastases. Most patients with primary brain tumors are treated to a total dose of 55–60 Gy delivered in 25–30 fractions. A variety of conformal strategies are used to reduce dose to remote areas of the brain. This contrasts with a total dose of 18–20 Gy used for CNS treatment of children with leukemia. Overall survival is improved with modern treatment techniques [1, 2], but patients still experience adverse late effects. Following fractionated whole-brain irradiation (fWBI), 50–90 % of long-term survivors (>6 months) have irreversible cognitive decline [3, 4]. The underlying molecular mechanisms that result in the loss of cognitive function after radiotherapy are not completely understood, and consequently, there is no treatment to prevent these adverse effects. Improving the quality of life of the growing population of patients who have received radiation treatment is an important objective.

WBI causes a number of deleterious cellular responses including neuronal dysfunction, blood–brain-barrier damage, astrocyte and microglia activation, and infiltration of peripherally derived monocytes [5–12]. WBI induces apoptosis of neural progenitor cells [8], which would be expected to affect overall cognitive function. Transplantation of neural stem cells in rodents can ameliorate radiation-induced cognitive dysfunction [13]. Neuronal injury and loss is not the only pathway contributing to cognitive deficits, since activation of non-neuronal cell types also affects overall brain function. We and others have shown that radiation induces infiltration of peripheral myeloid cells that depend on CCR2 signaling [14, 15] and that loss of the cytokine receptor CCR2 prevented the development of radiation-induced long-term cognitive deficits with no influence on neurogenesis [15]. Recently, Piao et al. demonstrated that oligodendrocyte progenitors derived from human embryonic stem cells can remyelinate the brain and rescue radiation-induced behavior deficits [16]. However, there is no clinically available agent that targets these convergent pathways in order to ameliorate or prevent permanent loss of cognitive function induced by WBI.

WBI induces up-regulation of pro-inflammatory cytokines and chemokines, including CCL2 [5, 17], which facilitates the recruitment of CCR2^+^ monocytes into the CNS. Following a single dose of 10 Gy WBI, deletion of CCL2 ameliorates deficits in hippocampal neurogenesis [18]. We demonstrated an increase of monocyte accumulation in the brain, as well as a decrease of microglia, 7 days following a single dose of 10 Gy WBI [5]. The recruitment of circulating monocytes into the CNS is regulated by the production of a number of soluble chemokines that interact with their cell surface receptors. One of these, colony-stimulating factor 1 receptor (CSF-1R), is a transmembrane tyrosine kinase receptor encoded by the *c-fms* proto-oncogene [19]. CSF-1/CSF-1R signaling regulates the survival, proliferation, chemotaxis, and differentiation of monocytes and macrophages [20–22]. Loss of CSF-1R results in complete elimination of microglia and severe monocyte deficits [23–25], and mice lacking CSF-1 have markedly reduced numbers of microglia [26].

Our group, and others, has used a single dose of WBI to model radiation-induced brain injury. However, in clinical treatment, virtually all patients receive fractionated brain irradiation with the goal of reducing toxicity to normal tissue. Here, we model the effects of fWBI in young adult mice by using a fractionated treatment paradigm (3 × 3.3 Gy) and explore the outcomes of CSF-1R blockade by PLX5622, analog of another CSF-1R inhibitor PLX3397 [27]. In other preclinical studies, PLX5622 has been used to diminish peripheral monocytes/macrophages [28, 29]. Similar to PLX3397, treatment with higher dose of PLX5622 (1200 ppm) depletes microglia in the CNS [28–34]. Recently, Dagher et al. showed that PLX5622 treatment (300 ppm) ameliorated cognitive deficits in aged Alzheimer’s mice [32]. In addition, our preliminary results (data not shown) suggest that lower (300 ppm) and higher (1200 ppm) doses of PLX5622 treatment achieved similar effect in reducing circulating monocytes in the periphery. In light of these results, we treated young adult mice with lower dose of PLX5622 (300 ppm) and evaluated cognitive outcomes at 1 month after fWBI, the earliest time point we see cognitive deficits in our hands. Our data show that fractionated brain irradiation, similar to single-dose irradiation, results in hippocampal-dependent memory deficits and loss of dendritic spine density in hippocampal granule neurons. Strikingly, CSF-1R blockade by PLX5622 can prevent memory deficits and dendritic spine density loss in mice treated with fWBI. Flow cytometry analyses of myeloid populations following treatment with PLX5622 demonstrate a strong correlation between improved cognitive performance and both decreased microglia numbers and monocyte accumulation in the brain. Using a clinically relevant model and pharmacologic approach, our data show that CSF-1R blockade by PLX5622 can prevent fWBI-induced cognitive deficits in mice by preventing loss of synaptic dendritic spines. These data implicate a new and therapeutically tractable role for infiltrating monocytes and microglia after brain irradiation in loss of synaptic function.

## Methods

### Compound

Control and PLX5622 (300 ppm formulated in AIN-76A standard chow, Research Diets, Inc.) chows were provided by Plexxikon Inc (Berkeley, CA). Approximately 1.2 mg of PLX5622 was ingested by each mouse per day (calculation based on 4 g/mouse chow daily).

### Animal procedures

All animal experiments were conducted in compliance with animal protocols approved by the Institutional Animal Care and Use Committee at the University of California, San Francisco (UCSF), following the National Institutes of Health Guidelines for animal care. C57BL/6J male mice were purchased from the Jackson Laboratory. CX3CR1^+/GFP^/CCR2^+/RFP^ animals were generated by crossing CX3CR1^GFP/GFP^/CCR2^RFP/RFP^ with C57BL/6J mice as previously described [5]. Starting at 8 weeks old, C57BL/6J mice were treated with PLX5622 or control chow for 21 days. Cranial irradiation started 7 days after drug treatment was initiated. Mice used for Golgi staining were euthanized at the end of the first NOR test (33 days after fWBI) were euthanized and perfused with ice-cold PBS, and the right hemispheres were used for Golgi staining. Mice used for flow cytometry analyses were euthanized at indicated time relative to the last day of fWBI.

### Radiation treatment

All the mice were anesthetized with intraperitoneal injection of a ketamine (100 mg/kg)/xylazine (10 mg/kg) mix and placed 16.3 cm from a cesium-137 source (JL Shepherd & Associates). The eyes and body were shielded by a lead collimator that limited the beam to a width of 1 cm. An extra lead plate was used to block exposure of the trachea. Irradiated groups received 1.65 Gy of irradiation on both side of the head to accumulate 3.3 Gy for each fractionated irradiation. Three fractions were delivered every other day over 5 days to accumulate a total dose of 10 Gy. Sham animals underwent the same procedures without radiation.

### Novel object recognition test

All the mice used for the NOR test were housed in a room with reversed light cycle (12 light/12 dark) for at least 2 weeks before tests. Tests were conducted during the dark cycle. The mice were handled 5 min each day for 5 days before habituation. An open arena (30 cm × 30 cm × 30 cm; L × W × H) was placed in a dimly lit behavior test room with an overhead camera. The mice were allowed to explore the open arena for 10 min for two consecutive days. On day 3, two identical objects were placed in the arena and mice were allowed to explore for 5 min. On day 4, one of the objects was replaced by a novel object and mice were allowed to explore for 5 min (Additional file 1: Figure S1A). Trials were recorded by the overhead camera and analyzed by an automatic video tracking system (EthoVision, Noldus) for movement tracking or by manual scoring for exploratory behavior. Exploratory behavior was defined as the animal directing its nose toward an object at a distance less than 2 cm. Objects were secured in the arena with magnets. Arena and objects were wiped with 70 % ethanol between trials to eliminate odor cues.

### Delayed matching-to-place dry maze test

Delayed matching-to-place (DMP) dry maze test was used to measure special working memory as described by Faizi et al. [35]. Briefly, we used a modified Barnes maze with 40 escape holes (*D* = 5 cm, 16 holes on the outer ring with 50-cm distance to the center, 16 holes on the middle ring with 35-cm distance to the center, and 8 holes on the inner ring with 20-cm distance to the center). All holes were uncovered with the exception of the escape hole, which is covered with a dark escape tube (a black PVC tube). The light was set to approximately 1200 lux, and a noise (2 kHz, 85 dB) was used during the test. Visual cues were placed on three sides of the maze. Mice were giving four trials each day with interval of 10 min. Mice were placed at the center of the maze under a dark box for 30 s. The trial started when the box was removed and ended when the mice found the escape hole within 90 s. Mice were guided to the escape hole by the experimenter if they could not find it within 90 s. Noise was turned off, and the escape hole was covered immediately after the mice entered. The mice were returned to their home cage after a 10-s delay. The maze surface and the escape tube were cleaned with 70 % ethanol (*v*/*v*) after each trial to minimize odor cues. The escape tube was kept at the same location and changed on each test days. Trials were recorded by an overhead camera and analyzed by Ethovision (Ethovision, Noldus).

### Metric distance test

Metric distance test was used to measure hippocampal function as previously described by Goodrich-Hunsaker et al. [36] with alterations to suit test in mice (Additional file 1: Figure S1E). Briefly, the test was performed on two consecutive days during the dark cycle with one habituation phase and 3 trials each day. On day 1, the mice were put into an open arena (30 cm × 30 cm × 30 cm, L × W × H) for 5 min. On trial 1, the mice were put into the arena with two identical objects placed at a distance of 28 cm to each other for 5 min. On trial 2, the mice were put into the arena with the same setting as trial 1 for 5 min. On trial 3, the distance between the objects was shortened to 14 cm and the mice were allowed to explore for 5 min. There was a 3-min interval between each trial, and the mice were put back into their home cage after each trial. One day 2, all trials were performed with the same setting as day 1 except that the distance between objects on trial 3 was changed to 21 cm. The objects and the arena were cleaned with 70 % (*v*/*V*) ethanol after each trial to minimize odor cues. Trials were recorded, and total time exploring the object pair was scored. Total time exploring during trial 1 on each test day was used as baseline of exploration.

### Flow cytometry

The mice used for flow cytometry analysis were euthanized and perfused with ice-cold PBS. The brains were removed and immediately placed into ice-cold HBSS. Brain samples were then dissociated using a Neural Tissue Dissociation Kit (P) (Miltenyi Biotec). Dissociated cells were resuspended in 10 ml of 30 % Percoll solution (Sigma) in an RPMI medium and laid over a 1 ml 70 % Percoll solution layer. After centrifugation at 800*g* for 30 min at 4 °C, interphase cells were transferred to a new 15-ml Falcon tube and washed with RPMI. Cell pellets were resuspended with FACS buffer (DPBS with 0.5 % BSA fraction V) and blocked with one volume of blocking solution (5 % normal mouse serum, 5 % normal rat serum, 5 % normal rabbit serum, 2 % FBS, and 1 % BSA fraction V in ×1 DPBS) for 30 min and stained for 30 min with fluorophore-conjugated antibodies on ice (CD45-BV711, CD11b-AF700, Ly6C-Pacific Blue, and Ly6G-PE were purchased from BD Pharmingen); 7AAD was used to exclude dead cells. Data were collected on an Aria III sorter (BD) and analyzed with FlowJo v10 software (Tree Star Inc.). At least 20,000 and 200,000 viable events were collected from each brain and blood sample, respectively.

### Golgi staining

Brain hemispheres stayed in Golgi staining solution (A Modified Golgi-Cox Stain for Neural Cells, Docket No. D4433, Cornell University) for 14 days and were transferred into 30 % sucrose in ×1 PBS overnight at 4 °C. The next day, tissues were transferred into fresh 30 % sucrose solution, protected from light and stored at 4 °C for at least 2 days. Brains were cut into 100 μm sections with a vibratome (VT1000 S, Leica, Wetzlar, Germany), mounted on gelatin pre-treated slides and dried for 2 days. Samples were then developed with the developing solution (A Modified Golgi-Cox Stain for Neural Cells, Docket No. D4433, Cornell University), covered, and dried. Images were taken on a Keyence 7000 system under a ×100 objective lens with immersion oil for hippocampal granule neurons. For each sample, 18–30 images were taken (2–3 images per section, 8–12 sections per mouse, *N* = 5–6 per treatment group) and used for dendritic spine density analysis. All protrusions from the dendrites were manually counted as spines regardless of morphology. A total length of at least 3000 μm of dendrites was analyzed from each animal using ImageJ (National Institutes of Health).

### Statistical analysis

NOR test results are shown as mean percentage of time spent on exploring each object (time exploring familiar or novel object/total exploring time) or mean discrimination index ((time exploring novel object − time exploring familiar object)/total exploring time) ± SEM. Metric distance test results are shown as percentage of time spent on exploring both objects in trial 1 on each test day. Results for DMP and metric distance tests were analyzed with ordinary two-way ANOVA with Bonferroni’s test for post hoc comparisons using day and experimental group as independent factors. Results for NOR test, dendritic spine density analysis, and flow cytometry with PLX5622 and fWBI treatments were analyzed with ordinary two-way ANOVA with Tukey’s test for post hoc comparisons using PLX5622 and fWBI as independent factors. Results for temporal analysis of monocyte accumulation were analyzed with one-way ANOVA with Bonferroni’s test for post hoc comparisons. All other comparisons between two sets of data were determined using *t* test. Error bars are shown as mean ± SEM. Details of each statistical analysis were described in figure legends. Graphs were analyzed and plotted with GraphPad Prism 6 software (GraphPad Software, Inc).

## Results

### Fractionated brain irradiation causes persistent hippocampal-dependent memory deficits

**Fig. 1:**
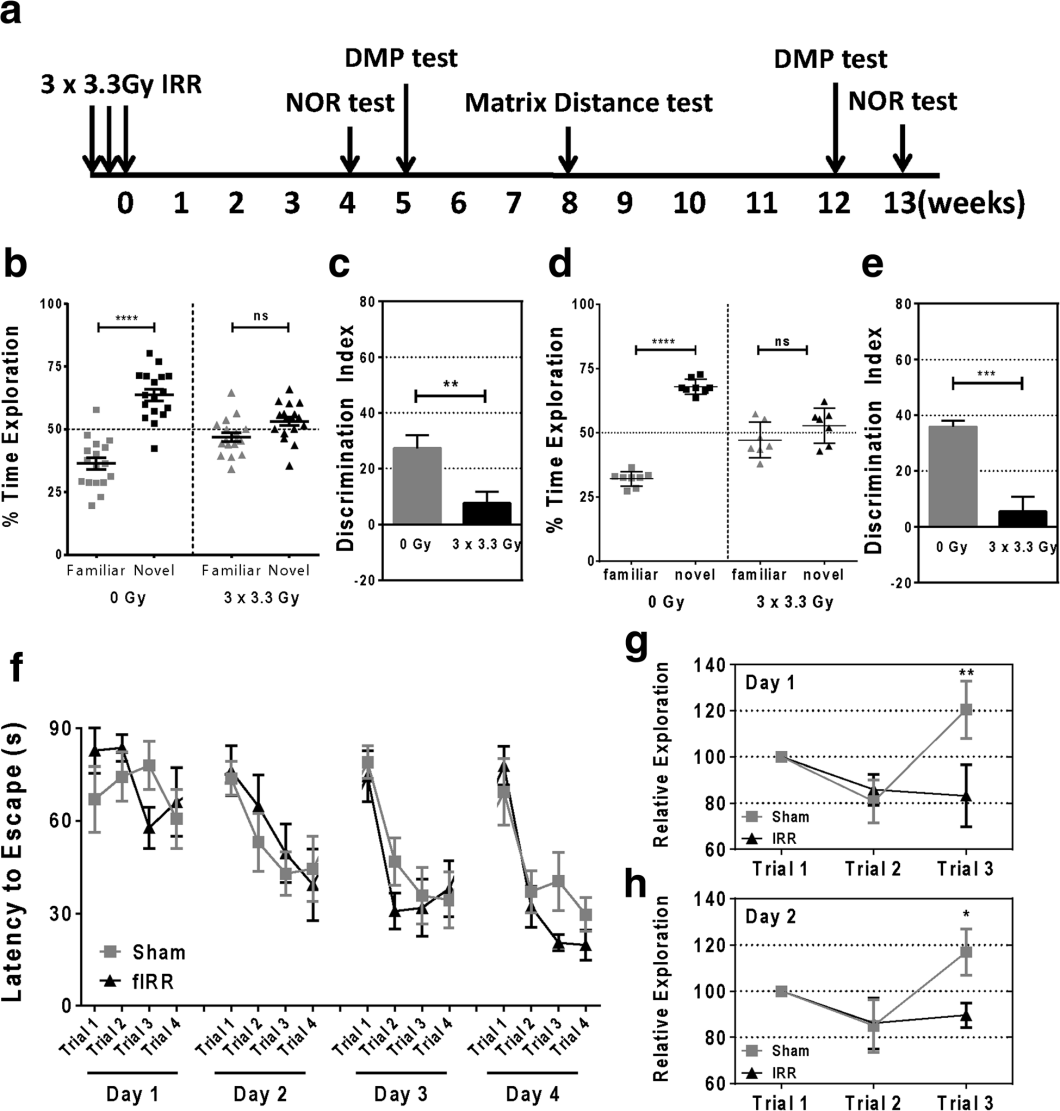
Fractionated whole-brain irradiation causes persistent hippocampal-dependent memory deficits. **a** Schematic charts of experiment timeline and cognition tests paradigm. Fractionated irradiation was delivered at 3.3 Gy/day every other day; NOR test was performed 4 and 13 weeks after the last radiation fraction; DMP tests were performed at 5 and 12 weeks after fWBI; matrix distance test was performed 8 weeks after fWBI. **b** Analysis of NOR at 4 weeks after fWBI (day 4 of NOR test, ****p < 0.0001, ns = not significant, n = 17, *t* test). **c** Discrimination index analysis for the NOR task (**p < 0.01, *n* = 17, t test). **d** Results of NOR at 13 weeks after fWBI (****p < 0.0001, ns = not significant, *n* = 7–8, *t* test). **e** Discrimination index analysis for the NOR test at 13 weeks post fWBI (***p < 0.001, *n* = 7–8, *t* test). **f** DMP test showed no impairment in working memory after fWBI (*n* = 7–8, two-way ANOVA). **g** Matrix distance test day 1, animals in the sham group but not in the irradiated group had significantly increased exploring time in trial 3 compared to trial 1 (**p < 0.01, *n* = 7–8, two-way ANOVA). (**h**) Matrix distance test day 2, only the animals in the sham group had significantly increased exploring compared to trial 1 (*p < 0.05, *n* = 7–8, two-way ANOVA)

The hippocampus, located in the medial temporal lobes of the brain, is responsible for memory formation. It has been suggested that the severity of cognitive impairment in humans depends upon the dose of radiation delivered to the hippocampus [37]. To determine the effects of therapeutically relevant fractionated doses of irradiation on hippocampal-dependent memory, adult male C57BL/6J mice were treated with three fractions of 3.3 Gy irradiation every other day to a total dose of 10 Gy and tested with a series of cognition tests at different time points (Fig. 1a). All mice tolerated fWBI and gained weight normally through the duration of study (data not shown). Hippocampal-dependent recognition memory was measured by the NOR test 4 and 13 weeks after the last fraction of radiation (Additional file 1: Figure S1A). In the 4 weeks’ NOR test, the mice in the non-irradiated group showed significantly higher preference toward the novel object (familiar = 36.32 ± 9.75 %; novel = 63.68 ± 9.75 %; *p* < 0.0001) while mice in the irradiated group had no preference toward either object (familiar = 46.82 ± 7.32 %; novel = 53.18 ± 7.32 %; *p* > 0.05. Fig. 1b). Consistent with these observations, the discrimination index was reduced in the irradiated group (*p* < 0.01) (Fig. 1c). There was no difference in distance traveled or total exploring time between the sham and irradiated groups (Additional file 1: Figure S1B and C). We observed similar results in the 13 weeks’ NOR test (Fig. 1d, 0 Gy familiar = 32.03 ± 2.89 %; 0 Gy novel = 67.97 ± 2.89 %, *p* < 0.0001; 3 × 3.3 Gy familiar = 47.22 ± 6.88 %; 3 × 3.3 Gy novel = 52.78 ± 6.88 %; and Fig. 1e, *p* < 0.001). The matrix distance test (Additional file 1: Figure S1E) was also used to evaluate hippocampal-dependent memory deficits 8 weeks after fWBI (Fig. 1a). Sham animals could detect both large and small changes in object locations and spent more time exploring the object pair in novel locations while irradiated animals failed to recognize these changes (Fig. 1g, *p* < 0.01, and Fig. 1h, *p* < 0.05). There was no significant difference in total exploring time during both test days (Additional file 1: Figure S1F and G). Working memory was measured by DMP test at 5 and 12 weeks after irradiation (Fig. 1a). We observed no significant difference in latency to the first escape hole between sham and irradiated animals at both time points (Fig. 1f and data not shown). There was no significant difference in velocity between sham and irradiated groups during the tests (Additional file 1: Figure S1D). Taken together, these results suggest that fractionated whole-brain radiation impairs hippocampal-dependent memory without affecting working memory in mice.

### CSF-1R blockade prevents hippocampal-dependent memory deficits after fractionated irradiation

**Fig. 2:**
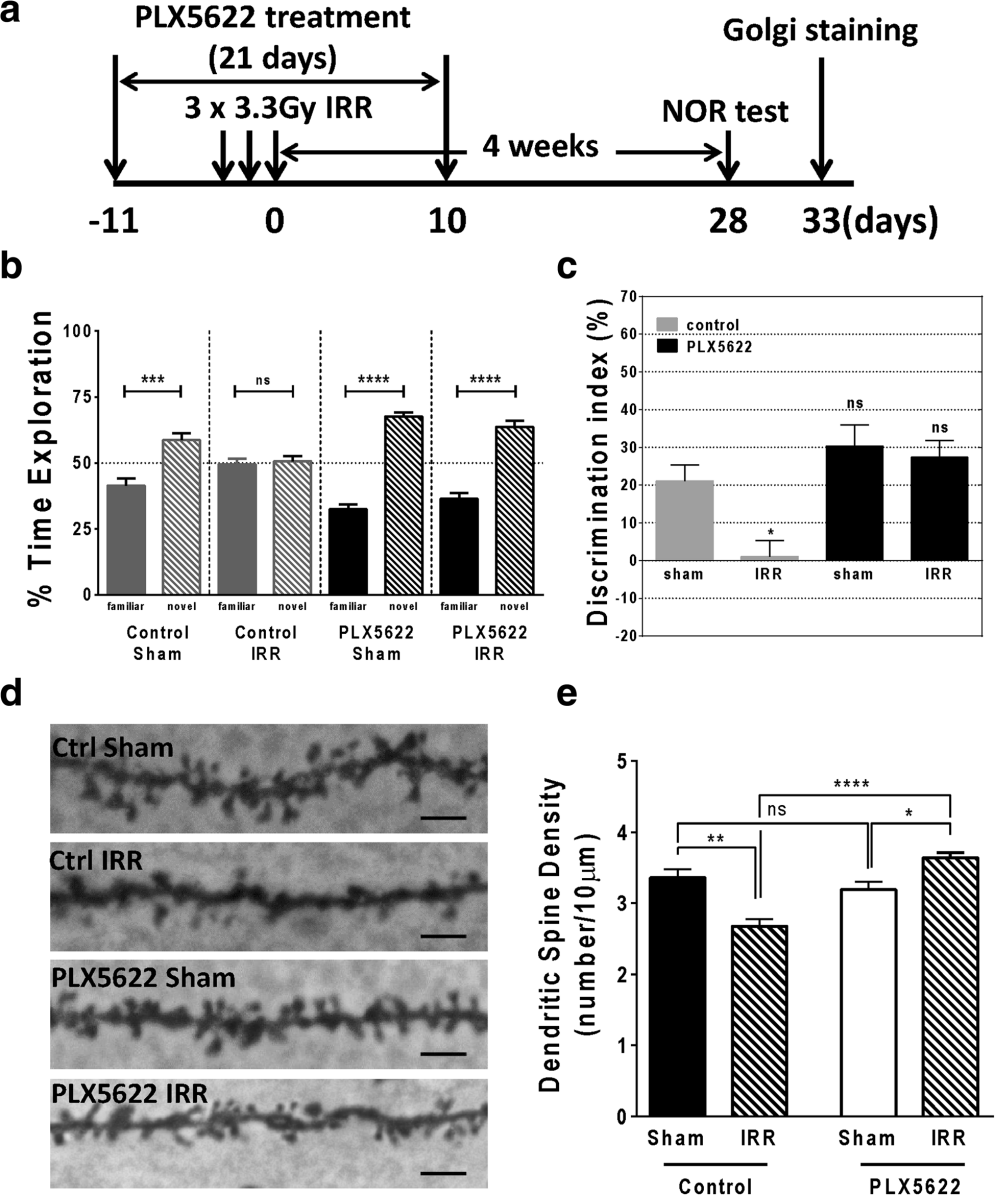
CSF-1R blockade prevents memory deficits and loss of dendritic spines after fractionated irradiation. **a** Schematic chart of experiment design. Mice were treated with PLX5622 7 days before the first radiation fraction and continued for a total of 21 days. NOR test was performed 4 weeks after the last radiation fraction. Mice were euthanized 1–2 days after the NOR test for Golgi staining. **b** Analysis of percentage time spent in exploring each object on test day (***p < 0.001, ****p < 0.0001, *n* = 11–12, *t* test). **c** Discrimination index analysis for the NOR task (*p < 0.05, ns = not significant, compared to control sham; F^PLX5622^ = 13.99, *p* = 0.0005; F^IRR^ = 5.825, *p* = 0.0201; F^Interaction^ = 3.198, *p* = 0.0808; *n* = 11–12, two-way ANOVA). **d** Representative images of dendrites from Dentate Gyrus granule neurons (×100, *scale bar* = 10 μm). **e** Comparison of dendritic spine densities (*p < 0.05, **p < 0.01, ****p < 0.0001, ns = not significant; F^PLX5622^ = 14.69, *p* = 0.0012; F^IRR^ = 1.287, *p* = 0.2715; F^Interaction^ = 29.81, *p* < 0.0001; *n* = 6, two-way ANOVA)

To investigate the effects of CSF-1R blockade on cognition after fWBI, we treated mice with PLX5622 (300 ppm) in chow for a total of 21 days, starting from 7 days before irradiation. The NOR test was performed 4 weeks after the last fraction of WBI (Fig. 2a). The bioanalysis of PLX5622 revealed that drug concentrations remained comparable throughout the treatment (Additional file 2: Figure S2A). As expected, animals on a control diet showed impaired memory after fractionated WBI (control sham: familiar = 41.38 ± 2.76 %; novel = 58.62 ± 2.76 %, *p* < 0.001; control IRR: familiar = 49.43 ± 2.12 %; novel = 50.57 ± 2.12 % *p* > 0.05) while PLX5622-treated mice showed no deficits (PLX5622 sham: familiar = 32.46 ± 1.70 %; novel = 67.54 ± 1.70 %, *p* < 0.0001; PLX5622 IRR: familiar = 36.32 ± 2.25 %; novel = 63.68 ± 2.25 %, *p* < 0.0001; Fig. 2b). The discrimination index analyses revealed a significant difference between the sham and irradiated groups on a control diet (control sham = 21.08 ± 4.33 %; control IRR = 1.145 ± 4.24 %; *p* < 0.05). Following PLX5622 treatment, there was no significant difference in the discrimination index between the sham and irradiated groups (PLX sham = 30.34 ± 5.67 %; PLX IRR = 27.37 ± 4.50 %; *p* > 0.05; Fig. 2c). There was no significant difference in total traveled distance or total exploring time among all experiment groups on the test day (Additional file 2: Figure S2B and C). These results demonstrate that treatment with PLX5622 for a brief period that precedes and follows irradiation can fully prevent fWBI-induced hippocampal-dependent memory deficits.

### CSF-1R blockade protects against dendritic spine loss after fractionated irradiation

Hippocampal neurogenesis is important for special and object recognition memory [38, 39]. Given the significant decrement in hippocampal-dependent memory induced by fWBI, we next sought to determine if the cognitive deficits were related to changes at the synaptic level in the hippocampus. We used Golgi staining to quantify dendritic spine density of granule cells in the dentate gyrus (DG) (Fig. 2d). We did not observe significant changes in dendritic spine densities at the end of PLX5622 treatment (day 10 post fWBI, Additional file 2: Figure S2D). However, at 33 days post fWBI, there is a significant reduction of dendritic spine density in irradiated animals (control sham = 3.359 ± 0.119/10 μm; control IRR = 2.676 ± 0.098/10 μm; *p* < 0.01). Strikingly, CSF-1R blockade with PLX5622 prevented dendritic spine density loss and resulted in increased dendritic spine density after fWBI (PLX5622 sham = 3.191 ± 0.115/10 μm; PLX5622 IRR = 3.638 ± 0.077/10 μm; *p* < 0.05). There was no significant difference between the control sham and PLX5622 sham groups (Fig. 2e, Additional file 3: Table S1).

### Fractionated WBI induces acute delayed monocyte accumulation in the brain

**Fig. 3:**
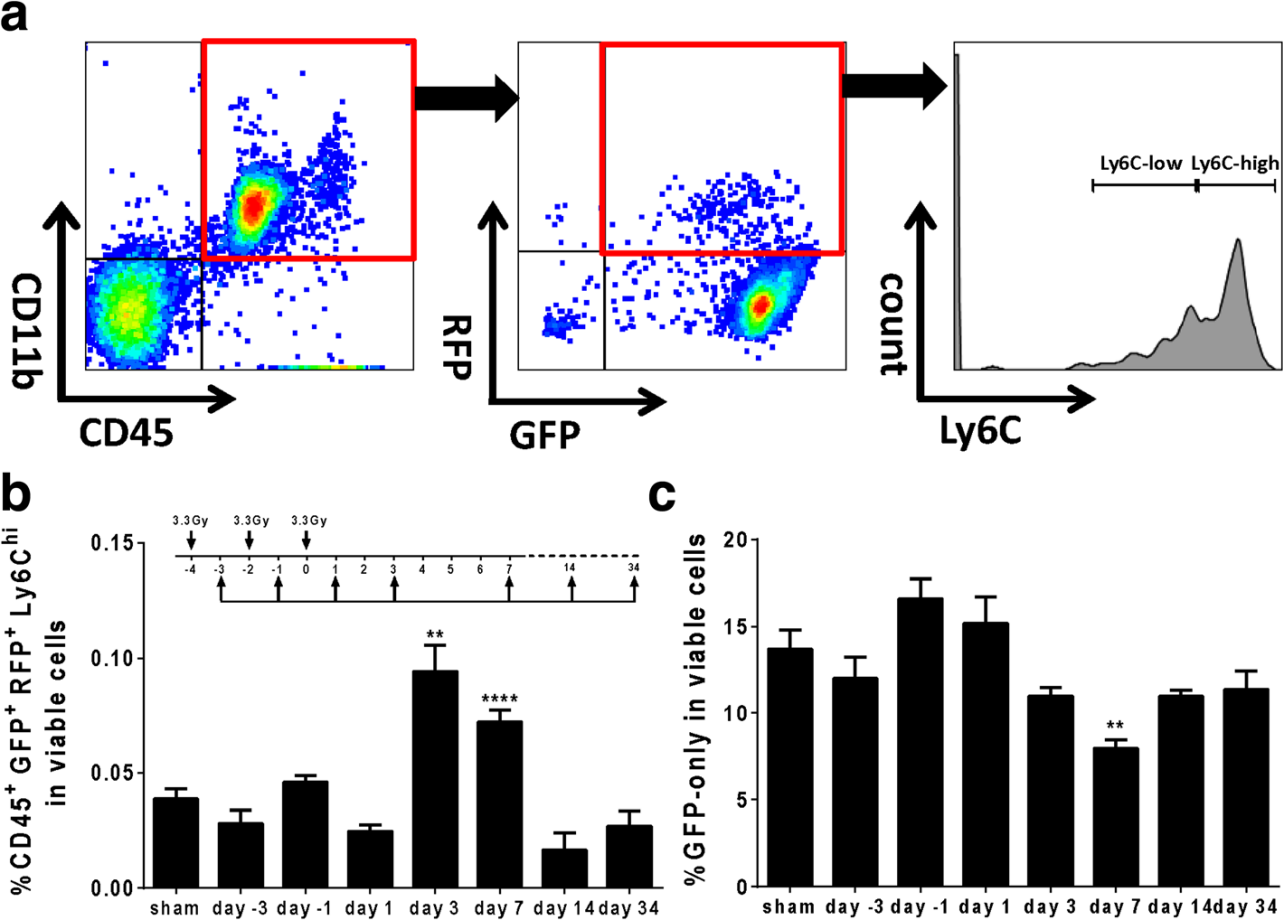
Fractionated irradiation induces inflammatory monocyte accumulation in the brain. **a** Representative images of gating strategy used in flow cytometry analyses. **b** Analysis showing temporal dynamics of inflammatory monocyte accumulation after fWBI (**p < 0.01, ****p < 0.0001, *n* = 4–5, one-way ANOVA). **c** Temporal dynamics of the GFP-only population in the brain after fWBI (**p < 0.01, *n* = 4–5, one-way ANOVA)

To determine the temporal dynamics of monocyte accumulation after fWBI, we used CX3CR1^+/GFP^CCR2^+/RFP^ reporter mice and flow cytometry analyses of the microglial and mononuclear cell populations. We first measured the numbers of Cd11b^+^GFP^+^RFP^+^ cells, which represent blood-derived monocytes [40] (Fig. 3a). There was no significant change in this population after each radiation fraction (sham = 0.095 ± 0.037 %, day −3 = 0.060 ± 0.013 %, day −1 = 0.098 ± 0.031 %, and day 1 = 0.070 ± 0.006) until 3 days after the last fraction (day 3 = 0.166 ± 0.029 %, *p* < 0.01), which recovered after day 7 (day 7 = 0.085 ± 0.022 %, *p* > 0.05, day 14 = 0.060 ± 0.004 %, day 33 = 0.090 ± 0.017 %. Additional file 4: Figure S3A). We observed a significant increase in the inflammatory monocyte population (CD45^+^GFP^+^RFP^+^Ly6C^high^ cells) at day 3 and day 7 after the last radiation fraction (sham = 0.039 ± 0.016 %, day −3 = 0.028 ± 0.012 %, day −1 = 0.046 ± 0.006 %, day 1 = 0.025 ± 0.006 %, day 3 = 0.094 ± 0.026 %, day 7 = 0.072 ± 0.011 %, day 14 = 0.017 ± 0.007 %, day 34 = 0.027 ± 0.007 %, Fig. 3b). There was no significant change in the CD45^+^GFP^+^RFP^+^Ly6C^low^ population (Additional file 4: Figure S3B). The population expressing only RFP (GFP^−^RFP^+^) remained unchanged throughout the experiment (Additional file 4: Figure S3C). The microglial population, represented by cells expressing only GFP (GFP^+^RFP^−^), remained unchanged until 7 days after the last radiation fraction, then declined by 35 %, followed by recovery after day 14 (sham = 13.68 ± 2.27 %, day −3 = 12.00 ± 2.45 %, day −1 = 16.58 ± 2.33 %, day 1 = 15.18 ± 3.029 %, day 3 = 10.97 ± 0.98 %, day 7 = 7.95 ± 1.15 %, day 14 = 10.97 ± 0.34 %, day 30 = 11.34 ± 1.11 %, Fig. 3c). Taken together, these results demonstrate that fractionated cranial irradiation induces Ly6C^high^ monocyte accumulation in an acute delayed pattern.

### CSF-1R blockade results in reduced blood monocytes and prevents monocyte accumulation in the brain after fWBI

**Fig. 4:**
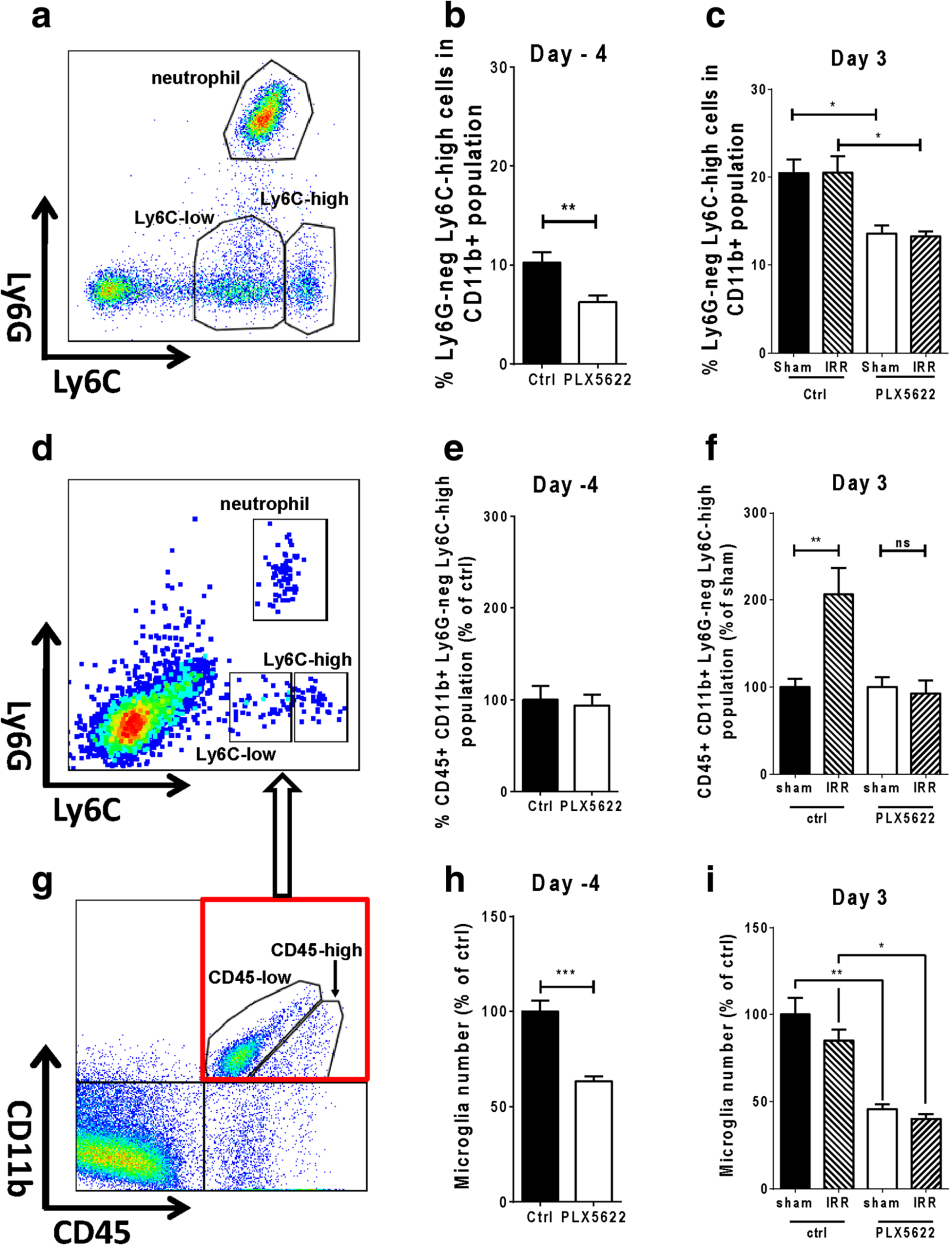
CSF-1R blockade reduces blood monocytes and prevents monocyte accumulation in the brain after radiation. **a** Gating strategy used in flow cytometry analyses for blood samples. **b** Comparison of blood monocyte between control and PLX5622-treated animals before irradiation (**p < 0.01, n = 8, *t* test). **c** Analyses of blood monocyte 3 days after the last radiation fraction (*p < 0.05; F^PLX5622^ = 24.97, *p* < 0.0001; F^IRR^ = 0.004, *p* = 0.95; F^Interaction^ = 0.007, *p* = 0.93; n = 6, two-way ANOVA). **d** Gating strategy used in flow cytometry analyses for brain samples. **e** Comparison of monocyte population in the brain between control and PLX5622-treated groups before irradiation (n = 8). **f** Analyses of brain monocytes 3 days after the last radiation fraction (**p < 0.01, ns = not significant, F^PLX5622^ = 9.334, *p* = 0.0045; F^IRR^ = 7.079, *p* = 0.0121; F^Interaction^ = 9.336, *p* = 0.0045; *n* = 9, two-way ANOVA). **g** Gating used to distinguish microglia from infiltrating myeloid cells (CD45-low vs CD45-high populations) (**h**) Comparison of microglia numbers before irradiation (***p < 0.001, n = 8, *t* test). **i** Analyses of microglia numbers 3 days after the last radiation fraction (*p < 0.05, **p < 0.01, F^PLX5622^ = 37.54, *p* = 0.0001; F^IRR^ = 1.602, *p* = 0.234; F^Interaction^ = 0.3323, *p* = 0.577; *n* = 9, two-way ANOVA)

CSF-1R is expressed in blood monocytes and is important for the survival, maturation, and differentiation of these cells [26, 41]. We analyzed blood samples from WT mice subjected to fWBI, and we observed a 35 % reduction of CD11b^+^Ly6G^−^Ly6C^high^ monocytes after 7 and 14 days of PLX5622 treatment (day −4: control = 10.23 ± 1.090 %, PLX5622 = 6.233 ± 0.671 %, *p* = 0.0102; day 3: control sham = 20.47 ± 1.562 %, PLX sham = 13.57 ± 0.967 %, *p* = 0.0106, control IRR = 20.50 ± 1.864 %, PLX5622 IRR = 13.27 ± 0.595 %, *p* = 0.0118; Fig. 4a–c). Importantly, there were no significant changes in Ly6C^low^ monocytes or neutrophils (Additional file 5: Figure S4A and B). These results suggest that the Ly6C^high^-expressing monocytes are susceptible to CSF-1R blockade while the Ly6C^low^-expressing monocytes are not affected. Further analysis of the brain samples revealed that PLX5622 treatment alone did not cause change in the numbers of Ly6C^high^ monocytes (Fig. 4d, e). Similar to results seen in CX3CR1^+/GFP^CCR2^+/RFP^ reporter mice, fractionated brain irradiation significantly increased Ly6C^high^ monocytes (ctrl sham = 100.0 ± 9.8 %, ctrl IRR = 206.4 ± 30.5 %, *p* = 0.0017, Fig. 4f). However, when treated with PLX5622 no significant difference was detected (PLX5622 sham = 100.0 ± 11.47 %, PLX5622 IRR = 92.65 ± 15.12 %, *p* > 0.05; Fig. 4f). There was no change in the numbers of CD45^+^CD11b^+^Ly6C^low^Ly6G^neg^ monocytes or CD45^+^CD11b^+^Ly6C^+^Ly6G^+^ neutrophils (Additional file 5: Figure S4 A and B). These results suggest that the inhibition of monocyte accumulation in the CNS by CSF-1R blockade is possibly due to reduced numbers of circulating monocytes in the blood.

### CSF-1R blockade results in decreased microglia in the brain

To determine the effects of CSF-1R blockade on microglia, we used flow cytometry analyses to compare numbers of microglia between animals treated with PLX5622 and control chows. We observed a significant reduction of microglia (CD11b^+^CD45^low^ cells, Fig. 4g) during PLX5622 treatment. Compared to the control groups, the PLX5622-treated groups had a 35 % reduction of microglial population at day −4 (7 days on treatment, Fig. 4h, *p* < 0.001) and 50 % reduction at day 3 (14 days on treatment; Fig. 4i, *p* < 0.01 ctrl sham vs PLX5622 sham; *p* < 0.05 ctrl IRR vs PLX IRR). Consistent with these observations, myeloid markers expressed in microglia were also reduced (Additional file 5: Figure S4C and D). However, microglia numbers fully recovered 4.5 weeks after PLX5622 withdrawal (data not shown). These results demonstrate that CSF-1R blockade by PLX5622 causes temporarily loss of microglia in the brain.

## Discussion

Radiotherapy is routinely delivered in fractions to treat cancers in order to reduce toxicity to normal tissues. However, most patients who receive WBI, or radiation to both temporal lobes, will develop cognitive deficits that can be profoundly disabling. Although conformal techniques and lowered CNS doses have reduced some adverse effects, there has been little research in the area of modifying the cellular response in the CNS following radiation, with the goal of reducing long-term cognitive deficits in patients. In this study, we modeled a clinical treatment paradigm by dividing 10 Gy WBI into three equal fractions delivered every other day and examined the cellular and behavioral consequences of that treatment. Our data show that fractionated brain irradiation results in hippocampal-dependent memory deficits and loss of dendritic spine density. CSF-1R blockade appears to rescue memory deficits and dendritic spine density loss in hippocampal granule neurons in the mouse model we have studied. Cumulatively, our findings offer novel insight into the mechanism of radiation-induced injury and demonstrate that the CSF-1R is a relevant and rational therapeutic target that could be used clinically to prevent irradiation-induced sequelae.

We previously reported that a single dose of 10 Gy WBI selectively disrupts hippocampal-dependent memory functions by affecting the cellular infrastructure responsible for plasticity and memory formation [13, 15]. Notably the hippocampus is exquisitely sensitive to WBI as non-hippocampal functions were intact [15]. Our current data demonstrate that fractionated radiation causes persistent cognitive deficits similar to those induced by 10 Gy WBI was delivered as a single dose. The NOR test examines hippocampal-dependent recognition memory for objects in rodents, and 1 month after irradiation, the mice had markedly impaired memory function that persisted up to 3 months. Further assessment of hippocampal dentate gyrus function with metric distance test showed significant impairment in animals that received fWBI. These cognitive changes are almost certainly the consequence of the activation of a diverse set of cellular responses that lead to synaptic alterations. Interestingly, higher cortical functions involved in episodic-like and working memory were not significantly affected by this irradiation paradigm. These data suggest while fractionation may reduce other types of radiation-induced normal brain injury, the hippocampus remains a selectively vulnerable structure.

Dendritic spines are postsynaptic components of excitatory synapses in the CNS. Their structural and density changes play fundamental role in synaptic functions, which are crucial for learning and memory [42, 43]. Decreased density and malformation of dendritic spines have been observed in neurodegenerative diseases, and loss of synapses is strongly correlated with cognitive decline [44, 45]. In the current study, we observed a decrease of dendritic spine density after fWBI comparable to what we and others previously reported using a single dose of 10 Gy WBI [46, 47]. These results suggest that fractionated irradiation routinely used to treat patients with primary brain tumors and metastases has substantial deleterious effects, similar to those observed following single-dose irradiation.

The exact mechanism of how ionizing radiation leads to impaired neuronal function, as demonstrated by the reduction of dendritic spines, is unclear. It is possible that some of this effect is directly related to DNA and cellular damage occurring in neurons following irradiation. However, there is also evidence to suggest that other cellular and molecular pathways activated after brain irradiation can contribute to neuronal dysfunction. Relevant to our study, analyses of myeloid cell populations reveal an accumulation of monocytes in the CNS after fWBI. CCR2 is a chemokine receptor expressed on cells of the myeloid cell lineage, and we have previously noted that CCR2 deficiency prevents radiation-induced hippocampal neuronal dysfunction from a cellular and behavioral perspective [15]. Based on these results, we postulated that inhibition of monocyte accumulation could prevent cognitive deficits induced by cranial irradiation. Because inhibition of CCR2 results in a more selective inhibition of myeloid cells, we chose to broadly block monocyte accumulation by treatment with PLX5622, a small-molecule selective CSF-1R inhibitor, which was tested in phase I clinical trials (ClinicalTrials.gov Identifier: NCT01329991 and NCT01282684). CSF-1R is essential for the survival and differentiation of macrophages and cells of the monocyte lineage [26, 41]. The association of increased macrophage infiltration with poor diagnosis in many types of cancers has led to some interest in targeting CSF-1R for cancer therapy [48], and studies have shown that CSF-1R inhibitors have anti-tumor effects [33, 49]. CSF-1R blockade has also showed efficacy in ameliorating other neuroinflammatory diseases. Gomez-Nicola et al. reported that blockade of CSF-1R with another tyrosine kinase antagonist inhibits microglia proliferation and slows neuronal damage in prion disease models [50].

Here, we irradiated young adult mice at 2 months of age (equivalent of 20 years of age in humans [51]) to reflect a population in human patients with longer survival and high risk to develop cognitive deficits after radiotherapy. We performed cognitive tests when mice were 3–5 months of age (equivalent of 24–30 years of age in humans [51]) to represent delayed time points when cognitive deficits are seen in humans. We demonstrate with flow cytometry analyses that PLX5622 treatment inhibits Ly6C^high^ monocyte accumulation in the brain after fWBI, possibly due to the reduced numbers of circulating Ly6C^high^ monocytes in the peripheral vasculature. We observed a 35–50 % decrease of microglia number in the brain during PLX5622 treatment, similar to the data reported by Dagher et al. with an Alzheimer’s model [32]. The numbers of brain microglia and peripheral blood monocyte both recovered after PLX5622 treatment was stopped, and PLX5622 treatment alone did not affect dendritic spine density or cognitive performance, suggesting that the effect of PLX5622 is transient and non-toxic. Ten days after fWBI, we observed a trend toward a decrease in dendritic spine density in both control and PLX5622-treated groups, but this did not reach statistical significance. However, at 33 days after fWBI, there was a statistically significant 20 % reduction of spine density in control groups, and CSF-1R blockade by PLX5622 treatment completely reversed the spine density loss. Given the limitations of Golgi staining and the manual scoring method used for dendritic spine counts [52], it is possible that we underestimated early-delayed structural and functional changes at 10 days post fWBI. Dye loading and electrophysiology techniques can be used in future studies to more accurately assess neuronal functions with CSF-1R blockade after fWBI. Nonetheless, our results are consistent with previous findings that suggest radiation-induced cognitive dysfunction is an ongoing process [3, 4]. We cannot, however, definitively conclude from our data whether the preserved dendritic spine density and cognition after fWBI are due to temporarily decreased microglia number or impaired monocyte accumulation during and immediately after fractionated brain irradiation. We observed a trend of better performance in the NOR test in PLX5622-treated animals compared to animals on control diet (Figs. 2b, c, PLX5622-sham vs control sham). However, given that PLX5622 specifically acts on the CSF-1R, which is expressed in myeloid cells, it is unlikely that PLX5622 have direct neurotrophic effects. It is possible that PLX5622 has secondary anti-inflammatory effects due to reduced microglia and monocyte numbers in the CNS during and after radiation. Further studies specifically targeting either cell population might help answer this question.

The duration of treatment required to modulate cognitive function is unclear. Several PPAR agonists and RAS blockers have been shown to be effective in ameliorating radiation-induced cognitive dysfunctions [53–55]. These studies all utilized continuous treatments starting from 3 to 7 days before radiation until the end of cognition assessment. In this study, we observe that transiently inhibiting the CSF-1/CSF-1R signaling reduces microglia number during radiation and blocks monocyte infiltration after radiation and is sufficient to ameliorate fWBI-induced neuronal and cognitive dysfunction. However, our previous report demonstrated that radiation-induced inflammation in the brain persists for at least 3 months after a single dose of 10 Gy cranial irradiation [15]. It is possible that dendritic spine loss in hippocampal granule neurons is a secondary effect caused by radiation-induced inflammation and is an ongoing process which lasts a substantial period of time. One limitation of our current study is that we only assessed dendritic spine density and cognitive performance up to 1 month after fWBI with PLX5622 treatment. Further studies are needed to determine if dendritic spine loss occurs at longer time points after brain irradiation, and whether temporary blockade of CSF-1R can permanently rescue this effect and ameliorate cognitive deficits. In addition, given the fact that PLX5622 treatment reduces a substantial portion of microglia, the safety, especially long-term effect of CSF-1R blockade, remains to be tested.

## Conclusions

In summary, we demonstrate that transient CSF-1R blockade by PLX5622 prevents fWBI-induced memory loss, which is associated with preservation of dendritic spine density of hippocampal neurons in the mouse model studied. Therefore, targeting CSF1R signaling could provide a possible approach to prevent incidence and severity of irradiation-induced brain injury.

## Additional files

Additional file 1: Figure S1. (A) Experimental paradigm for NOR test. Mice were allowed to explore the arena for 10 minutes during the habituation phase (Day 1 and Day 2). On the training phase (Day 3), mice were allowed to explore the arena for 5 minutes with two identical objects. On the test phase (Day 4), mice were allowed to explore the arena for 5 minutes with one familiar object and a novel object. (B) There was no difference in total time spent exploring both the familiar and the novel objects between sham and irradiated groups. (C) There was no difference in distance traveled during the test phase (Day 4) between sham and irradiated groups. (D) There was no difference in velocity throughout the DMP test (n = 8). (E) Experimental paradigm for matrix distance test. On each day each animal went through a 5-minute habituation phase followed by three 5-minute trials. The distance between two identical objects were kept at 28 cm during trials 1 and 2, and changed during trial 3. On day 1, objects were placed with a distance of 14 cm; on day 2, objected were placed with a distance of 21 cm. (F) and (G) No difference was detected in total time exploring across trials or groups (n = 8). (TIF 436 kb) Additional file 2: Figure S2. (A) Bioanalysis of PLX5622 in the blood and brain. Radiation does not cause increased drug accumulation in the brain (n = 6). (B) There is no difference in travel distance among groups (n = 11-12). (C) There is no difference in total exploring time among groups (n = 11-12). (D) Comparison of dendritic spine densities 10 days after fWBI (21 days on PLX5622 treatment) shows no significant difference among groups (n = 5–6). (TIF 238 kb) Additional file 3: Table S1. Dendritic spine quantification of granule neurons in dentate gyrus.(XLSX 14 kb) Additional file 4: Figure S3. Temporal analyses of periphery derived cells in the brain during and after fWBI. (A) Periphery derived monocytes/macrophages (Cx3cr1^+^Ccr2^+^) changes over time. Significant increase was observed on 3 days after the last radiation fraction (**p* < 0.05). (B) Cx3cr1^+^Ccr2^+^Ly6C^low^ monocytes changes over time. No significant differences were observed. (C) RFP-only population changes over time. n = 4–5. (TIF 255 kb) Additional file 5: Figure S4. PLX5622 treatment does not cause changes in Ly6C^low^ monocytes or neutrophil but results in reduced expression of myeloid cell markers in the brain. (A) Flow cytometry analysis of Ly6C^low^ monocytes 3 days after last radiation fraction (n = 6). (B) Flow cytometry analysis of neutrophils 3 days after the last radiation fraction (n = 6). (C) Analysis of qPCR results of myeloid markers between control and PLX5622 treated groups before fWBI (**p* < 0.05, n = 6). (D) qPCR analyses of myeloid markers 3 days after the last radiation fraction (**p* < 0.05 ctrl_sham vs PLX_sham, #*p* < 0.05 ctrl_IRR vs PLX_IRR, n = 6). (TIF 444 kb)

## Abbreviations

CSF-1R: Colony-stimulating factor 1 receptor
fWBI: Fractionated whole-brain irradiation
CNS: Central nervous system
