# Supplementary figures and images for: Colony-stimulating factor 1 receptor blockade prevents fractionated whole-brain irradiation-induced memory deficits
Source: J Neuroinflammation. 2016 Aug 30;13(1):215. doi: 10.1186/s12974-016-0671-y (PMC5006433; doi:10.1186/s12974-016-0671-y)

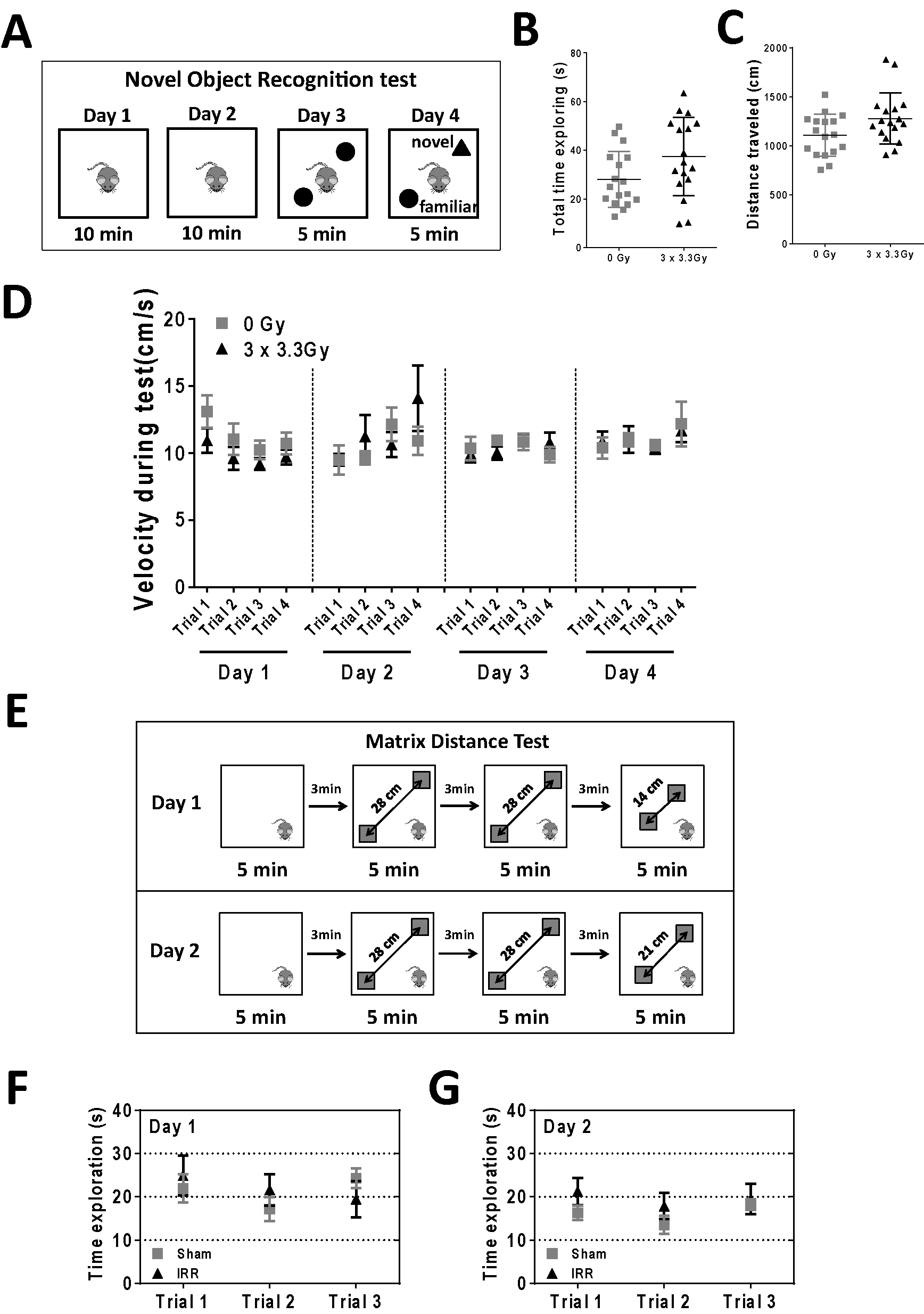

Supplement: Additional file 1: Figure S1. — (A) Experimental paradigm for NOR test. Mice were allowed to explore the arena for 10 minutes during the habituation phase (Day 1 and Day 2). On the training phase (Day 3), mice were allowed to explore the arena for 5 minutes with two identical objects. On the test phase (Day 4), mice were allowed to explore the arena for 5 minutes with one familiar object and a novel object. (B) There was no difference in total time spent exploring both the familiar and the novel objects between sham and irradiated groups. (C) There was no difference in distance traveled during the test phase (Day 4) between sham and irradiated groups. (D) There was no difference in velocity throughout the DMP test (n = 8). (E) Experimental paradigm for matrix distance test. On each day each animal went through a 5-minute habituation phase followed by three 5-minute trials. The distance between two identical objects were kept at 28 cm during trials 1 and 2, and changed during trial 3. On day 1, objects were placed with a distance of 14 cm; on day 2, objected were placed with a distance of 21 cm. (F) and (G) No difference was detected in total time exploring across trials or groups (n = 8). (TIF 436 kb) [file 12974_2016_671_MOESM1_ESM.tif]

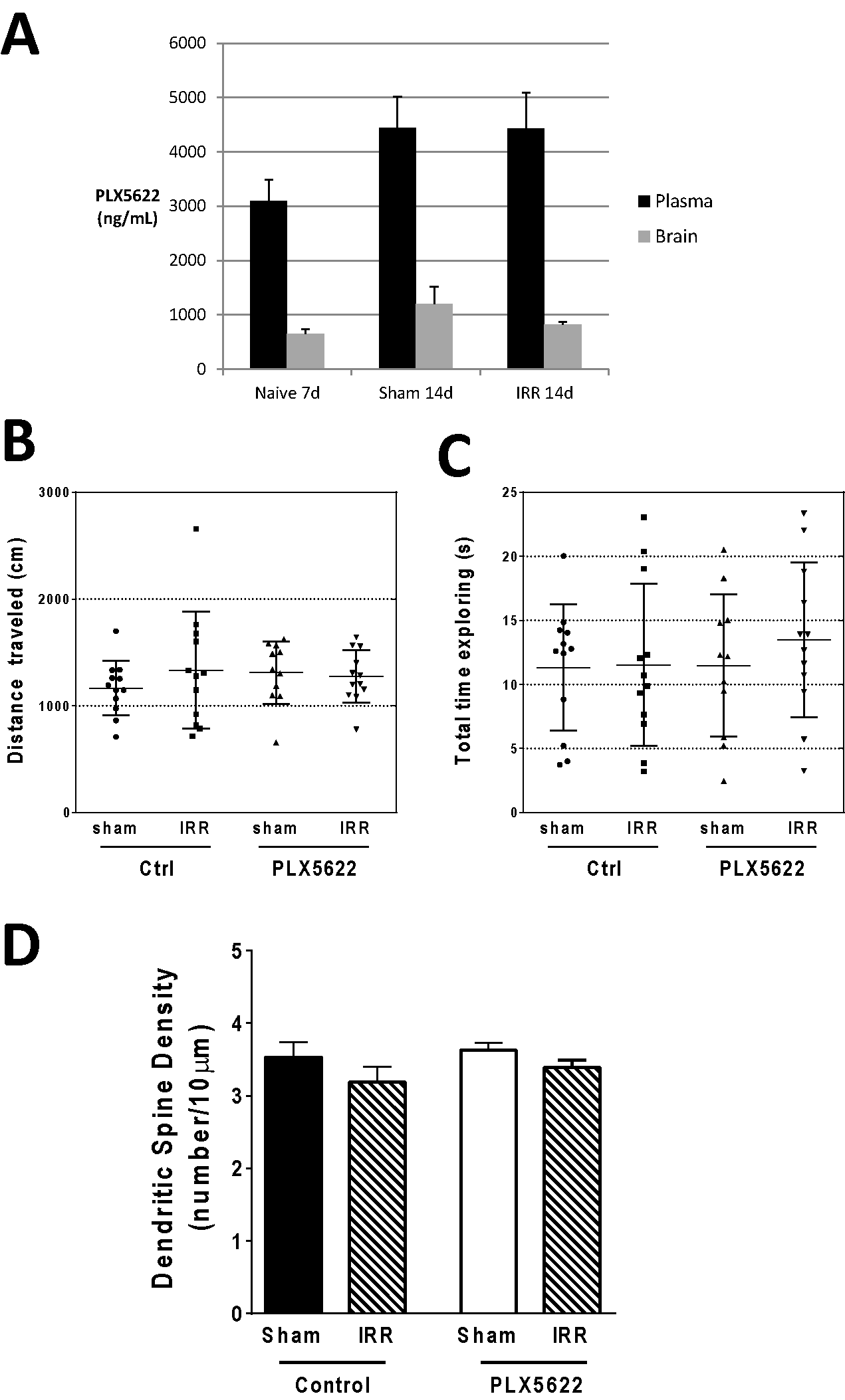

Supplement: Additional file 2: Figure S2. — (A) Bioanalysis of PLX5622 in the blood and brain. Radiation does not cause increased drug accumulation in the brain (n = 6). (B) There is no difference in travel distance among groups (n = 11-12). (C) There is no difference in total exploring time among groups (n = 11-12). (D) Comparison of dendritic spine densities 10 days after fWBI (21 days on PLX5622 treatment) shows no significant difference among groups (n = 5–6). (TIF 238 kb) [file 12974_2016_671_MOESM2_ESM.tif]

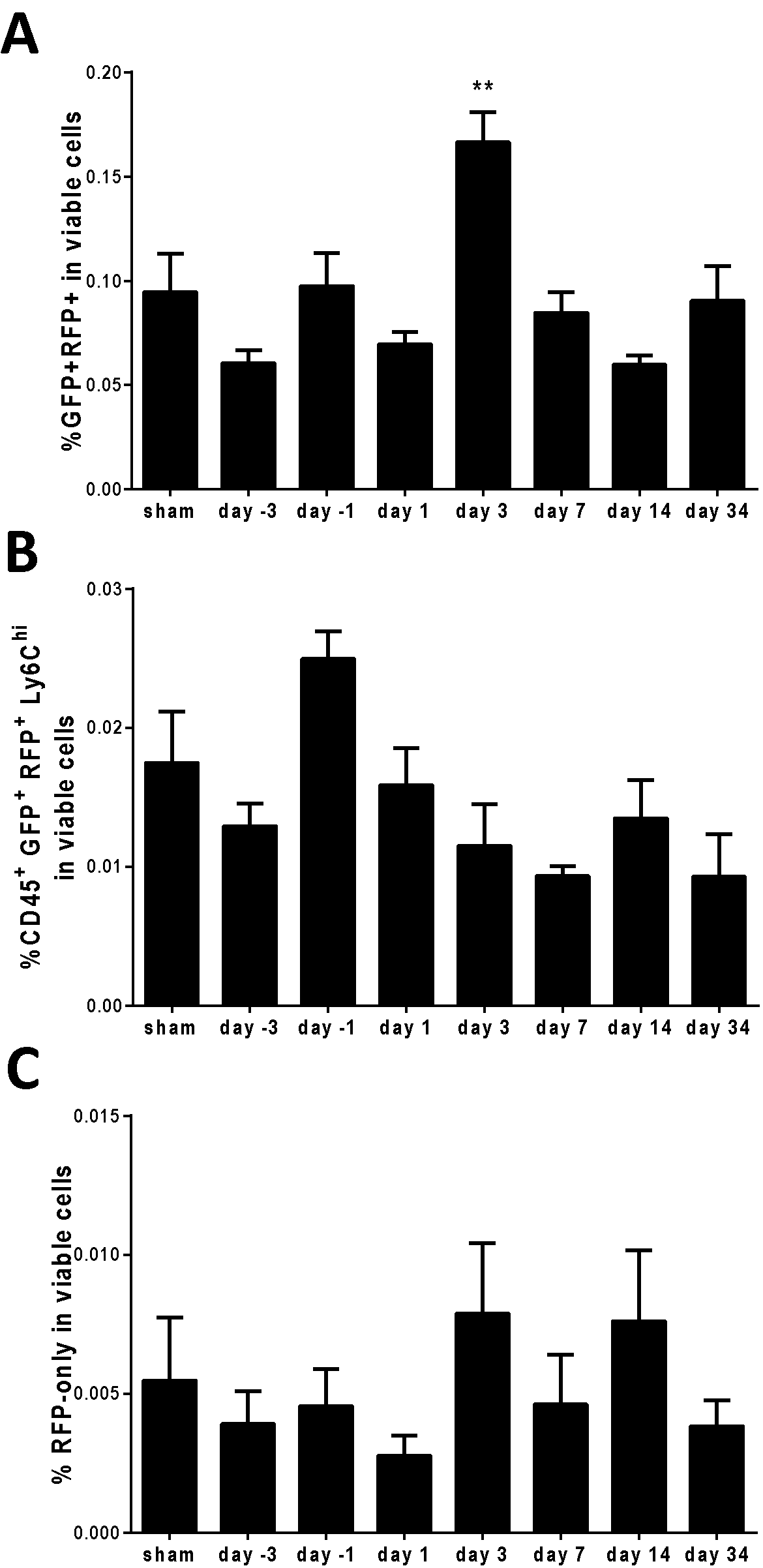

Supplement: Additional file 4: Figure S3. — Temporal analyses of periphery derived cells in the brain during and after fWBI. (A) Periphery derived monocytes/macrophages (Cx3cr1+Ccr2+) changes over time. Significant increase was observed on 3 days after the last radiation fraction (*p < 0.05). (B) Cx3cr1+Ccr2+Ly6Clow monocytes changes over time. No significant differences were observed. (C) RFP-only population changes over time. n = 4–5. (TIF 255 kb) [file 12974_2016_671_MOESM4_ESM.tif]

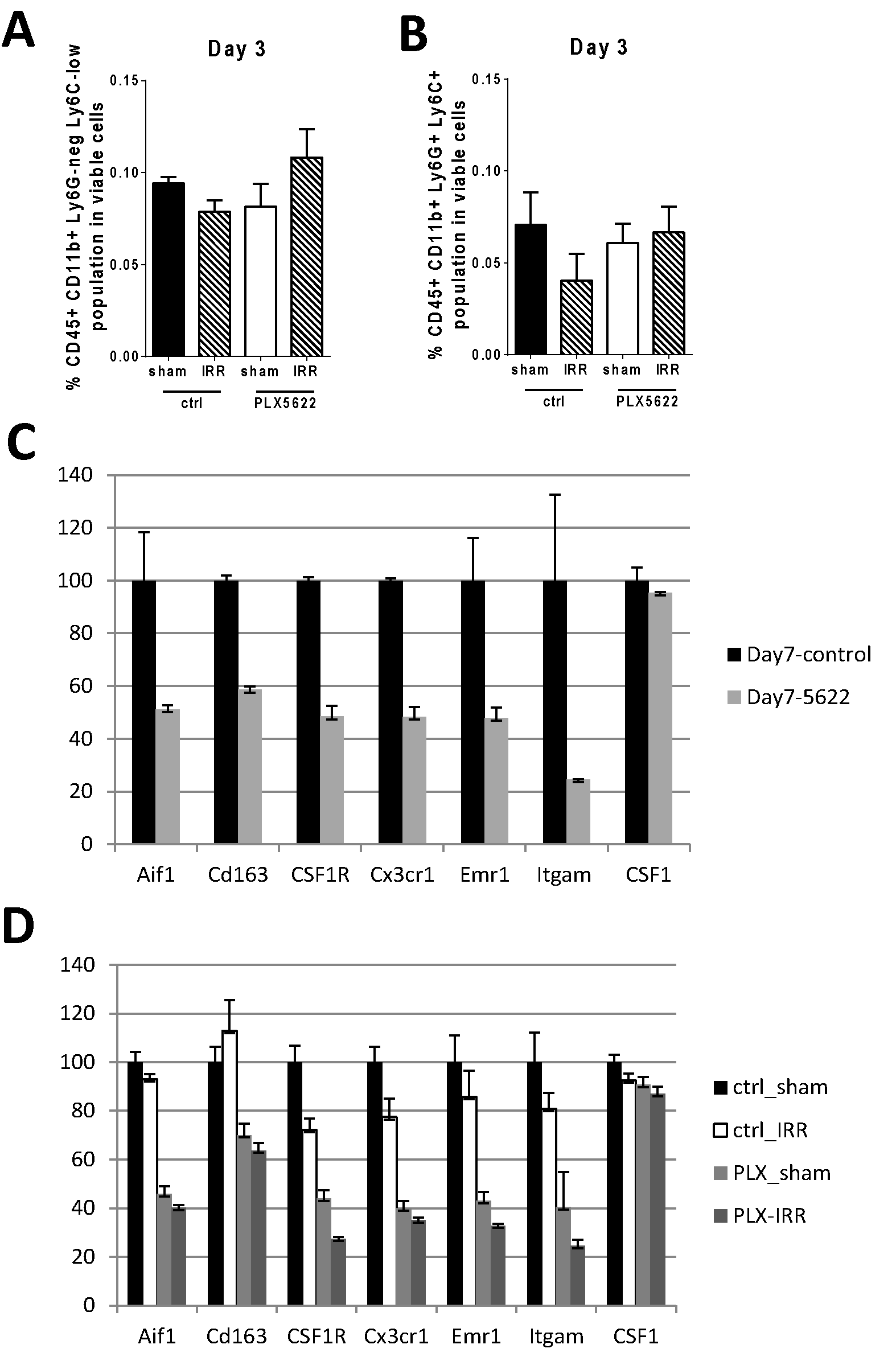

Supplement: Additional file 5: Figure S4. — PLX5622 treatment does not cause changes in Ly6Clow monocytes or neutrophil but results in reduced expression of myeloid cell markers in the brain. (A) Flow cytometry analysis of Ly6Clow monocytes 3 days after last radiation fraction (n = 6). (B) Flow cytometry analysis of neutrophils 3 days after the last radiation fraction (n = 6). (C) Analysis of qPCR results of myeloid markers between control and PLX5622 treated groups before fWBI (*p < 0.05, n = 6). (D) qPCR analyses of myeloid markers 3 days after the last radiation fraction (*p < 0.05 ctrl_sham vs PLX_sham, #p < 0.05 ctrl_IRR vs PLX_IRR, n = 6). (TIF 444 kb) [file 12974_2016_671_MOESM5_ESM.tif]
